# Perspective: Framework for Developing Recommended Intakes of Bioactive Dietary Substances

**DOI:** 10.1093/advances/nmab044

**Published:** 2021-05-07

**Authors:** Allison A Yates, Johanna T Dwyer, John W Erdman, Janet C King, Barbara J Lyle, Barbara O Schneeman, Connie M Weaver, serving as an ad hoc Working Group on a Framework for Developing Recommended Intakes for Dietary Bioactives

**Affiliations:** Agricultural Research Service/USDA (Retired), Johnson City, TN, USA; Department of Medicine, Tufts University School of Medicine and Jean Mayer USDA Human Nutrition Research Center on Aging at Tufts University, Tufts Medical Center, Boston MA, USA; Department of Food Science and Human Nutrition, University of Illinois, Urbana, IL, USA; Department of Nutritional Sciences and Toxicology, University of California, Berkeley, CA, USA; B Lyle, Inc., Deerfield, IL, USA; Northwestern University, School of Professional Studies, Evanston, IL, USA; Department of Nutrition, University of California, Davis, CA, USA; Weaver & Associates Consulting, LLC, Colorado Springs, CO, USA; Department of Nutrition Science, Purdue University, West Lafayette, IN, USA

**Keywords:** dietary reference intakes, dietary supplements, food sources, diet and health, systems for nutrition evidence reviews, dietary bioactives, recommended intakes, reference values

## Abstract

Dietary bioactives are food substances that promote health but are not essential to prevent typical deficiency conditions. Examples include lutein and zeaxanthin, omega-3 fatty acids, and flavonoids. When quality evidence is available, quantified intake recommendations linking dietary bioactives with specific health benefits will enable health professionals to provide evidence-based information to consumers. Without evidence-based recommendations, consumers use information from available sources that often lack standards and rigor. This article describes a framework to develop guidance based on quality evidence fully vetted for efficacy and safety by qualified experts, and designed to communicate the amounts of specific dietary bioactive compounds with identified health benefits. The 4-step Framework described here can be adapted by credible health organizations to work within their guideline development process. Standards of practice used in clinical guidelines are adapted to quantify dietary bioactive intake recommendations from foods consumed by the general public, by taking into account that side effects and trade-offs are often needed for medical treatments but are not acceptable for dietary bioactives. In quantifying dietary bioactive recommendations, this Framework establishes 4 decision-making steps: *1*) characterize the bioactive, determine amounts in specific food sources, and quantify intakes; *2*) evaluate safety; *3*) quantify the causal relation between the specific bioactive and accepted markers of health or normal function via systematic evidence reviews; and *4*) translate the evidence into a quantified bioactive intake statement. This Framework provides a working model that can be updated as new approaches are advanced.

## Teaser

**Statement of Significance**: A credible step-by-step process for translating evidence into quantified dietary bioactive intake recommendations is provided; the approach is relevant to nutrition guidance developers globally.

## Introduction

Many working definitions for bioactives exist, depending on the purpose and to some extent the regulatory constructs in which they are considered (1). The Framework described in this article for quantifying dietary bioactive intake recommendations draws on the US National Institutes of Health, Office of Dietary Supplements working definition that bioactives are “constituents in foods or dietary supplements, other than those needed to meet basic human nutritional needs, that are responsible for changes in health status” (2). A few examples of dietary bioactives include the carotenoid lutein/zeaxanthin, various flavonoids, and bioactive peptides, all of which are present in commonly consumed foods. The process used by the Food and Nutrition Board (FNB) of the Institute of Medicine (IOM), now under the National Academies of Sciences, Engineering, and Medicine (NASEM), to develop dietary reference intake (DRI) values for nutrients employs a framework based on the relations between nutrient exposure and biological/clinical indicators of adequacy (typically to prevent deficiency), excess, or reduction of chronic disease risk (3, 4). A similar construct is lacking for developing evidence-based recommendations for safe and effective intakes of bioactives that have broader effects promoting health, rather than primarily preventing deficiency or decreasing chronic disease risk. The Framework in this article addresses this gap by providing a process based on quality evidence from systematic evidence reviews fully vetted by qualified experts, which can lead to recommended quantified intakes from food of specific dietary bioactive compounds with identified health benefits.

Quantifying intake recommendations for bioactives differs from doing this for nutrients with established DRIs, which typically are well defined chemically, with well-characterized metabolic roles in specific health outcomes. In contrast, most bioactives are chemically complex and diverse, and their role or effects on health can be partially met at times by other very similar chemical constituents, making their individual contributions to specific health outcomes or status often difficult to ascertain. Some bioactives can be rapidly converted into other active or nonactive constituents through the processes of digestion, absorption, and metabolism. Therefore causal inference associating a quantified intake of a bioactive with a benefit to normal structure/function or disease risk reduction can be more complex than that which occurs with nutrients.

This Framework provides a step-by-step approach to quantify bioactive intake recommendations in food forms when the quality of evidence for benefit is determined to be sufficient and there is little or no evidence of harm. Although generally similar in approach, the Framework differs from medical treatment guideline development in which decisions need to be made, even when the quality of the evidence showing efficacy is relatively low or there are potentially negative treatment effects. In contrast, it is not imperative to recommend intakes for a dietary bioactive for which there is no overt medical condition requiring treatment, and thus intake recommendations for bioactives can be dichotomous: yes/no. This dichotomous approach eliminates the "weak" recommendations and "strong" designations used in the GRADE (Grading of Recommendations Assessment, Development, and Evaluation) approach.

This Framework does not apply to bioactives consumed in supplement forms due to safety concerns associated with bioactives when isolated and consumed in concentrated forms (5). Different matrices (e.g., as liquid, powder, pill, gummies, or others), conditions of use, manufacturing processes, and other issues can have an impact on the safety and efficacy of dietary supplements. Therefore, bioactives provided in the form of supplements need to be reviewed on a product-by-product basis for dose, chemical form and matrix, and ADME (absorption, distribution, metabolism, excretion), and the constituents under study typically require standard toxicity testing. This Framework provides a step-by-step approach to quantify bioactive intake recommendations specifically in food forms, where the quality of evidence for benefit is determined to be sufficient, and there is little or no evidence of harm.

## Overview of the Framework

This approach describes a 4-step process for evaluating the evidence about a dietary bioactive with demonstrated benefit to human health (i.e., supporting normal structure, function, or reducing risk of acute or chronic conditions) and, where it is warranted, translating it into a quantified intake range for generally healthy populations or subpopulations. When recommended, intakes are issued in a formatted summary statement reflecting the quantified range of intake for a dietary bioactive consumed in food forms. The following principles apply when recommendations are made for dietary bioactives:

- Recommended dietary bioactive intakes are based on specific health outcomes using the best available evidence; multiple health measures of the outcome may be considered.
- Recommended ranges are expected to change as new data become available at differing levels of intake, or with new findings regarding benefit(s) to health.
- Recommended intake ranges are limited to consumption levels without known adverse effects that have the potential to harm an individual.
- Food forms include those in which the bioactive is naturally present or enhanced as long as it meets regulatory requirements. If data from isolated extracted forms are used to inform recommendations, chemical (species) and physical form (matrix) must be relevant to those consumed in food forms.

This Framework builds on the 2017 FNB report *Guiding Principles for Developing Dietary Reference Intakes Based on Chronic Disease*, which outlines an approach to characterize a quantitative relation between a nutrient or other food substance (termed NOFS) and specifically chronic disease risk (4) (see **Box A**). Notably, the FNB report addresses bioactives only within the context of reducing the risk of chronic disease (thus not including outcomes related to maintaining normal structure, function, or health maintenance), which reflects the FNB committee's charge to address chronic disease specifically (4).

Box A: Principles applied to this Framework from the report *Guiding Principles for Developing Dietary Reference Intakes Based on Chronic Disease* (4)

- Extrapolate intake-response data for chronic disease (now termed chronic disease risk reduction, or CDRR) only to populations similar to the studied populations.
- Provide an intake *range* rather than a single number to express CDRR recommendations.
- If changes in disease risk occur only at intake levels above the upper level (UL), CDRR is unnecessary, because the UL should not be exceeded.
- Ensure that the level of certainty for the intake-response relationship to chronic disease risk is at least *moderate* using the Grading of Recommendations, Assessment, Development, and Evaluation (GRADE) system.
- Conduct a risk/benefit analysis when benefits and harms overlap for nutrients or other food substances (NOFS) and chronic disease risk. The method used to characterize and decide on the balance should be explicit and transparent.

### A variety of health indicators can be valid outcome measures

For this bioactive Framework, outcome measures refer to normal structures or functions, health status, and disease risk reduction. Some health measures include maintaining normal or improving postprandial blood glucose, macular pigment density (a structure that supports visual function), cognitive performance, BMI, blood lipids, and blood pressure. Valid and reliable measures of normal health status along with disease risk reduction biomarkers accepted by authoritative decision-makers can be used to quantify the health benefits of dietary bioactives. Such biomarkers are described in the IOM report on the *Evaluation of Biomarkers and Surrogate Endpoints in Chronic Disease* (6).

### Establishing a causal relation between intake of a bioactive and health outcome is challenging but necessary

Double-blind, randomized clinical trial interventions evaluating a pure bioactive are the most direct means of establishing a causal relation between the bioactive and a specific health outcome. However, if the bioactive is part of a habitual diet, particularly if present in multiple foods, it can be impractical or impossible to design a study to test the bioactive independent of the baseline diet or matrix. In that case, the quantity consumed cannot be ascribed solely to the effect of that intervention unless the other nontested dietary sources are controlled for adequately. Also, it should be noted that when a semipurified extract or purified bioactive compound is evaluated, it might not behave the same when consumed in isolation rather than in a food matrix (with altered bioavailability being a notable difference). Therefore, although evidence from blinded, randomized interventions with isolated extracts provides important information, it should be recognized that observational studies can also provide highly relevant supporting evidence.

### Food composition data on the bioactive are critical to estimating intakes and developing recommended ranges

Slight changes in chemical and physical structure of a bioactive compound from one food matrix or type of food to another can affect its bioavailability or physiological effect. As an example, polyphenols occur in several chemical subclasses that differ by food source, including variations in glycosylation, esterification, and polymerization, which can affect bioavailabilty (7). Therefore, food composition data for bioactives should provide detailed information on the various chemical structures so that dietary intakes can be estimated. Steps for developing food composition databases for bioactive components have been summarized (8). The flavonoid database Phenol-Explorer 3.0 is an example of a comprehensive database containing >35,000 content values for 500 polyphenol compounds in >400 foods; additionally, the USDA special database provides values for flavonoid aglycones in selected food items (9, 10). Although food composition data for other bioactives are published in the literature, up-to-date, publicly available food composition databases documenting other bioactive substances are critically needed to advance research on health effects as well as ultimately to translate intake recommendations into food choices. Without adequate information on food composition and thus dietary intakes, the impact of dietary intakes on health outcomes cannot be determined.

### Recommendations can apply to the general population or a specific subpopulation

**FIGURE 1:**
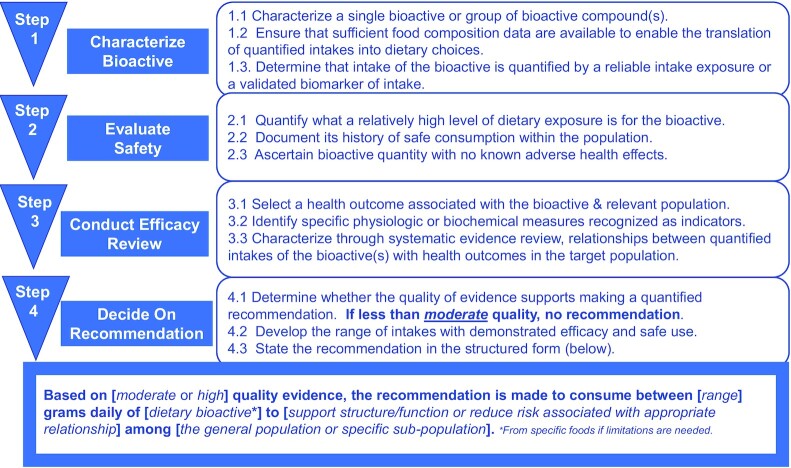
Sequential Framework to evaluate potential health benefits of dietary bioactives. Each step of the Framework must be completed before the review moves forward to the next step. If, in completing Step 4, the decision is to make a recommendation, the format of that recommendation follows the wording of the statement in the final box.

Recommendations based on a body of evidence that is considered to be generalizable to the overall population will have broad reach. However, if evidence is not relevant to the general population, then the subpopulation of interest should be specified at the onset of the process to ensure recommendations are developed within the context of the specified subpopulation. This is consistent with the 2017 FNB guiding principles report for setting DRIs based on risk reduction for chronic disease, which stipulates that recommendations should only be applied to populations with the disorder or conditions that are similar to studied populations (4). Overall, the quantified range of the dietary bioactive intake recommendation should reflect the evidence from which it is based and be stated in the structured recommendation summary statement (**Figure 1**). For example, if evidence is based exclusively on adult populations, then an expert working group should decide whether and how to quantify intakes for children, just as it should for any other subgroup not directly relevant to the evidence base. Extrapolation beyond the population represented in the evidence base should be the exception and should be done only with a clearly stated rationale.

## Stepwise Decision-Making Process to Develop Bioactive Recommendations

This section describes a sequential decision-making Framework to be used in reviewing evidence and developing bioactive intake recommendations that quantify a range reflecting both efficacy and safety (Figure 1). The process results in a structured format summary statement developed by experts free of financial, intellectual, and professional conflicts of interest. The Framework is specific in terms of the key steps and principles to be used in decision-making, but allows health organizations to adapt the approach to their own guidance process. Note that all of the substeps within each step in the Framework (e.g., 1.1, 1.2, 1.3 in Step 1) must be met in order to proceed to the next step. The narrative associated with each step describes the main principles to apply in decision-making.

### STEP 1: Characterize the bioactive, determine amounts in food sources, and quantify intakes

Characterize a single bioactive or group of bioactive compound(s) associated with a specific health outcome by chemical structure or isolation techniques.
Ensure that sufficient food composition data are available to enable the translation of quantified intakes into dietary choices.
Determine that intake of the bioactive is quantified by a reliable intake exposure or a validated biomarker of intake.

#### Form and source affect bioactivity

In elucidating causal relations between bioactive dietary components and specific health outcomes it is important to know the variety of chemical forms and matrices in which they are found. The matrix, chemical form, and dose, as well as individual variation in metabolism based on genetic and gut microbial factors, can all affect the ADME of the parent compound or its metabolites. For example, a systematic review indicated that the flavan-3-ols present in tea (epicatechins) improve flow-mediated dilation in blood vessels, whereas the flavan-3-ols in cocoa/chocolate (catechins) improve flow-mediated dilation as well as blood pressure (11). This evidence suggests that both forms and matrices are consistent with reducing risk of cardiovascular disease, but can differ with respect to blood pressure specifically. In this example, it would be possible to focus on either specific compounds within the class (e.g., epicatechins) or on a broad number of compounds within the class (e.g., all flavan-3-ols combined). Physiological effects on health outcomes drive the decision of how broadly or narrowly (e.g., broadly reduce risk of cardiovascular disease or specifically blood pressure) to define the dietary bioactive associated with the specific outcome. Therefore, it is important to define and appropriately combine (or not) bioactives based on the chemical structures and food matrices in this first step.

#### Identifying forms and occurrence in foods

The unique chemical structure(s) related to the identified role in health must be measured in various foods with a high degree of confidence (accurate and reproducible), including under various conditions of preparation (e.g., cooking, drying, freezing, or canning). Such information on the bioactive's composition in foods is necessary to quantify the relation between the intake of the bioactive with possible health outcomes as well as to quantify intakes with an established history of safe consumption. Additionally, food composition data are necessary for health professionals to translate recommended quantities into food-based dietary advice for consumers. If bioactive food composition data are lacking or weak, the process of developing recommendations must wait until sufficient food composition data are available.

#### Determining intake of bioactive(s)

When bioactive intakes are calculated using dietary intake reports combined with food composition databases, substantial information on the methodology used to estimate intake should be made available. This includes describing how the approach corrects for random error, uses designs that decrease subject bias, and estimates usual intakes by repeated measures that take into account seasonal variation as well as under- or overreporting.

Biomarkers of intake are necessary when evidence for the possible relationship between the bioactive and a health benefit is exclusively from observational or other human studies lacking measured, quantified total bioactive intake (e.g., an intervention study in which baseline diets contribute varying amounts of the bioactive). Validated biomarkers are expected to show a precise and reproducible dose–response relationship with known amounts of the biomarker from an inclusive list of foods typically consumed by differing age groups under various metabolic conditions, and be applicable across ethnic groups and gender groups.

#### Estimating intakes from classes of bioactive compounds

In some cases, multiple chemical structures are represented in a class of bioactive components (e.g., the class of flavan-3-ols includes both monomers such as epigallocatechin gallate, and polymers such as proanthocyanidins and theaflavins). It is necessary that the main components be chemically/structurally identified, and that analytical methods exist to quantify individual components. Where structural diversity exceeds current analytical capacity (e.g., proanthocyanidins or oxidized flavan-3-ols in tea and cocoa), quantitative estimates of their content in foods should be provided using the best analytical approaches available.

If validated biomarkers of intake or exposure exist and are validated in similar population groups to those of interest, then such biomarkers of intake can be used to quantitatively describe its relation to a function or other health parameter. When intake biomarkers correlate with the group of bioactive components, the effects should be attributed only to the specific compounds, and not generalized to a group or class of compounds. For example, when blood concentrations of specific long-chain omega-3 fatty acids are related to health outcomes, the evidence cannot be extended to all dietary omega-3 fatty acids as a single combined category.

There is also the possibility that the active component that produces a health benefit includes more than a single metabolite, rather than the bioactive itself. Therefore, it is useful to use established nomenclature, such as has been recommended for polyphenol catabolites (12). Similar to single-compound bioactives, substantial information on methods used to estimate intake must be available before attempting to quantify the relation between intake and benefit.

### STEP 2: Evaluate safety

Quantify what a relatively high level of dietary exposure is for the bioactive.
Document its history of safe consumption within the population.
Ascertain the bioactive quantity with no known adverse health effects.

#### How safety concerns are addressed

Intake quantities recommended for dietary bioactives must be consistent with a history of safe consumption to ensure that the recommended range is not associated with known adverse effects that significantly impact health. Note that the criterion of no known adverse health effects excludes modest usual day-to-day general discomfort that occurs when eating a varied diet or making changes in one's diet (e.g., transient changes in stool consistency or other minor day-to-day digestive signs or symptoms). If long-term dietary intake data among typically “high” consumers are unavailable, standard toxicological testing should be used to quantify safe levels, particularly if it appears that benefits accrue when consumed at levels above historical safe use. [Note that Step 2 is inappropriate for documenting safety of specific commercial products/ingredients because individual product characteristics matter and approval regulations vary globally. In other words, this step is not intended to substantiate individual product safety testing in which specific form, format, use level, use, as well as other factors apply. Many of these factors are noted, for illustration purposes, in the 2016 US FDA guidance related to assessment and notification of new dietary ingredients (13).]

Information on safety should take into account the nutritional, botanical, and toxicological peer-reviewed scientific literature, reports from authoritative bodies such as government expert groups or those of regulatory bodies, survey data of food or nutrient composition and consumption, and other peer-reviewed material describing the composition of the sources of the bioactive, as well as proprietary information where available from survey or consumption data, product sales data, and compositional analyses.

Where information is available on adverse health effects of specific bioactives, including that found in adverse event reporting systems or published studies, it must be reviewed and evaluated by experts trained in toxicology, biostatistics, and data analysis. The experts will determine if no quantified intake recommendations should be considered, or, if they are made, whether they should carry explicit conditional statements. The guiding principles for determining unreasonable risk articulated in the IOM/National Research Council report on *Dietary Supplements: A Framework for Evaluating Safety* provide a useful construct for the purposes of this Framework on dietary bioactives (a brief excerpt provided in **Box B** highlights key points for illustrative purposes) (14).

Box B: Excerpt on evaluating data to determine unreasonable risk from *Dietary Supplements: A Framework for Evaluating Safety*, adapted by substituting dietary bioactive terminology in place of original wording as noted by italics (Adapted from reference 14 with permission)

Guiding Principles for Evaluating Data to Determine Unreasonable Risk:

General principles

- Absence of evidence of risk does not indicate that there is no risk.
- Proof of causality or proof of harm is not necessary to determine unreasonable or significant risk.
- Integration of data across different categories of information and types of study design can enhance biological plausibility and identify consistencies, leading to conclusions regarding levels of concern for an adverse event that may be associated with consumption of a *dietary bioactive*.

Human data

- A credible report or study finding of a serious adverse event in humans raises concern about the *bioactive component's* safety and requires further information gathering and evaluation; final judgment, however, will require consideration of the totality of the evidence.
- Historical use should not be used as prima facie evidence that the *bioactive component* does not cause harm.
- Considerable weight can be given to a lack of adverse events in large, high-quality, randomized clinical trials or epidemiological studies that are adequately powered and designed to detect adverse effects.

Animal data

- Even in the absence of information on adverse events in humans, evidence of harm from animal studies is often indicative of potential harm to humans.

Related substances

- Scientific evidence for risk can be obtained by considering if the plant constituents are compounds with established toxicity, are closely related in structure to compounds with established toxicity, or the plant source of the botanical *bioactive component* itself is a toxic plant or is taxonomically related to a known toxic plant.
- *Bioactive components* that are endogenous substances or that may be related to endogenous substances should be evaluated to determine if their activities are likely to lead to serious effects. Considerations should include the substance's ability to raise the steady-state concentration of biologically active metabolites in tissues and whether the effect of such increases would be linked to a serious health effect.

In vitro data

- Validated* in vitro studies can stand alone as independent indicators of risk to human health if a comparable exposure is attained in humans and the in vitro effects correlate with a specific adverse health effect in humans or animals.

*In this report, in vitro assays are considered validated when their results have been proven to predict a specific effect in animals and/or humans with reasonable certainty (not necessarily universally accepted or without detractors).

#### Determining quantity of a bioactive intake with a history of safe use

The highest quantified level of bioactive intake for which there is a history of consumption with no known significant adverse health effects needs to be determined so that the final recommended quantity reflects efficacious levels with demonstrated safe use. History of use must be relevant to the dietary bioactive forms and sources included in the evaluation. In this Framework, which pertains *only* to bioactives in food forms, history of safe use of the bioactive is primarily from foods. If data are augmented with supporting data from bioactives in dietary supplement forms, it should be noted that history of bioactive use from supplements is probably of relatively much shorter duration and the conditions of use can be quite different (including consumption of bolus doses without other foods or beverages). Choice of a subpopulation for quantifying history of high use should reflect long-term high use relevant to the population for which the recommendation is being quantified. This promotes harmony in recommendations across similar populations. For example, tea consumption in populations with high intakes is useful in quantifying high intakes for geographic regions with comparable diets and populations, such as tea intake in Turkey for the Middle East, Ireland for Western Europe and the United States, and China for Asia.

The method used by the US FDA can be followed to determine the quantity of a bioactive with a history of safe use (13). In brief, the evaluation needs to characterize and compare the identity of the bioactive with what has been historically consumed, and then describe how the composition of the bioactive and consumption levels relate to the safe intake for the bioactive compound(s). The evaluation should consider:

- dose (intake per serving),
- total daily intake at the mean and high exposure (90th percentile) levels among those who ingest the bioactive,
- duration of use,
- conditions of use,
- frequency of use of the historically consumed food source, and
- characteristics of the populations consuming it, such as age, health status, etc.

The US FDA considers 25 y of widespread use as the minimum for establishing a history of safe use, along with the number of consumers who consumed the ingredient (in this case the bioactive), and the frequency of consumption (13). This construct of what constitutes a history of safe use is a principle that applies in this bioactive Framework, not a regulatory requirement pertaining to specific product approval. Alternatively, if some other well-established regionally relevant standard for history of safe use exists, then experts evaluating a bioactive intake recommendation could apply those regionally relevant standards supported with a well-documented rationale.

Because source affects bioactivity, the source should be specified when quantifying historically safe consumption, because processing can alter not only the food matrix, but also the compounds present and the bioactive's potency itself. If the dietary bioactive is present in a widely consumed food(s), the quantity of the bioactive historically consumed establishes a reasonable expectation of safety as long as there are minimal differences in dose, composition, processing, conditions of use, and the target population. However, safety assessment and testing apply even if there is a history of use when the food contains other compounds with known or suspected adverse effects, has reported or published adverse effects, or has a limited history of use.

#### Applying standard toxicology testing: when and how

By definition “dietary bioactives” are present in the diet and therefore generally have a long and well-substantiated history of being consumed safely in food. However, experimental safety testing applies when:

- the dietary bioactive compound or food source has known or suspected adverse effects (e.g., copresence of factors with known/suspected adverse effects), or
- historical bioactive intake differs significantly from levels evaluated for health benefit, such as relatively higher intakes, longer duration, or
- the recommended bioactive is targeted to a different population group than the population with long-term historical use.

When toxicology testing is appropriate, it is useful to review all toxicological or clinical studies (published or proprietary) in which the test substance is identical to the bioactive under review, the matrix is the same, the route of consumption is the same, and the dosage and duration are similar to what is proposed. Established state-of-the-art toxicology testing guidelines from an authoritative body applicable in the region for which bioactive intakes are being recommended should be followed to establish safety. As an example relevant to the United States, current guidance from the FDA on new dietary ingredients defines specific genotoxicity, oral tolerance, reproductive effect, and teratogenicity tests (13). Toxicology testing should reflect the most up-to-date relevant standards applicable to the region in which quantified bioactive intakes are under consideration for recommendations.

#### Ascertaining safety

Experienced food safety experts should evaluate the history of safe use (or when necessary, safety testing), in order to prepare a concise statement indicating the highest quantity of the bioactive with no known serious adverse health effects, and provide supporting documentation. This level is used in Step 4 to ensure that the recommended range of bioactive intake based on demonstrated efficacy is at or below what is determined to be the safe level.

### STEP 3: Quantify the relationship between the bioactive(s) and accepted markers of health or normal function via systematic evidence reviews

Select a health outcome associated with the dietary bioactive and relevant population.
Identify specific physiological or biochemical measures recognized as indicators of the health outcome.
Characterize through systematic evidence review, relationships between quantified intakes of bioactive(s) with health outcomes in the target population.

#### How to consider health outcome(s) associated with the bioactive of interest

##### Human data demonstrating causal relations provide the primary evidence

In Step 3, human data providing evidence of a causal relationship guide the decision on which health outcome to select, including measures related to normal structure or function, indicators of maintaining health, or reduced risk of disease. Animal and cell culture studies are appropriate for determining the potential mechanism and site of action, but are insufficient to quantify levels of dietary bioactives for specific human health outcomes. Identifying specific mechanism(s) of action strengthens confidence in the bioactive's relationship with a function/health outcome or chronic disease process, but is not necessary to make a recommendation where there is substantial evidence that the relation is causal. Primary evidence of an independent, causal relation between the bioactive(s) and health comes from human intervention studies. A descriptive summary of the evidence available in the scientific literature (sometimes referred to as knowledge mapping) is useful to determine if the evidence available from human intervention trials with supporting observational studies warrants proceeding with the process to develop recommended intakes (15). Knowledge maps on dietary bioactives such as lutein/zeaxanthin and flavan-3-ols are illustrative (16, 17).

##### Considerations regarding measures as indicators of health outcome

Experts in the relationships between the bioactive and health outcomes under evaluation are critical decision-makers when selecting meaningful health outcomes relevant to the bioactive. For most health outcomes, a specific validated and reliable physiological, structural, functional, or biochemical measure is used to establish a causal link between the dietary bioactive and the health outcome. An example of a biochemical indicator is macular pigment optical density (MPOD). MPOD is a noninvasive measure of basic eye structure associated with visual function, including photo stress recovery, glare disability, and contrast sensitivity (18). Therefore, in evaluating the role of the bioactive compounds lutein/zeaxanthin (which comprise the primary pigments in the macula), MPOD serves as a meaningful indicator of eye structure associated with normal visual function. An example of physiological function is endothelium-dependent vasodilation related to healthy blood flow, which has been recognized as such by the European Food Safety Authority, commonly referred to as EFSA (19).

Measuring chronic disease outcomes directly has limited feasibility because disease can take years to manifest. Therefore, qualified biomarkers of chronic disease risk are important outcome measures. They are defined as biomarkers that can impact the risk of chronic disease development, and for which the time course for the effect is known, as is the proportion of the population affected if intakes are inadequate or adequate (4).

Surrogate end points (i.e., clinically qualified biomarkers of risk) can be considered for the specific bioactive under review but only if they are in the causal pathway (4). For example, the US FDA accepts certain validated surrogate end points for establishing health claims (e.g., serum total and LDL cholesterol and blood pressure for cardiovascular disease, bone mineral density for osteoporosis, adenomatous colon polyps for colon cancer, and elevated blood glucose and insulin resistance for type 2 diabetes) (20). Because regional differences can exist in recognized surrogate end points relevant to the population for which the quantified bioactive intakes are intended, disease surrogate end points should be recognized by authoritative scientific bodies within the intended geographic region. If a surrogate clinical measure is not yet recognized as a validated surrogate for disease risk but is used, the rationale for its validity along with uncertainties should be identified and discussed.

##### Approaches to managing multiple relevant health outcomes

Multiple health measures related to the selected outcome should be included when evaluating evidence. For example, a recommended bioactive intake to support general cardiovascular health can be evaluated by multiple types of specific measures such as blood pressure and vascular plasticity, clinical measures, or disease incidence. Supporting normal vision can include more than a single indicator of visual function such as visual acuity and contrast sensitivity, or the status of specific structures of the eye. Furthermore, relevant outcome measures might differ by subpopulation (e.g., maintaining cognitive health in older adults could be based on different measures than the indicators used to measure cognitive development in children).

A summary of the evidence available in the scientific literature is a useful tool to inform decision-makers regarding the specific physiological measures by subgroups, before proceeding to a systematic evidence review (15). GRADE and the USDA Nutrition Evidence Systematic Review (NESR) are among currently accepted systematic evidence review methods (21–23).

It is important to remember that because of the multifactorial nature of diseases, beneficial effects of a bioactive on intermediate markers are neither synonymous with nor a guarantee of disease prevention. For example, improved flow-mediated dilation can improve vascular health but cannot be assumed to be sufficient to prevent cardiovascular disease. Intakes of dietary bioactives should be communicated within the context of supporting normal structure/function and reducing risk of (not necessarily preventing) disease conditions.

#### Conducting the evidence review

##### Critical factors to consider in conducting the evidence review

A systematic evidence review of the association between a bioactive and a health outcome is necessary (not simply a general or narrative expert review). The evidence review should be published in a peer-reviewed publication and completed by experts in systematic reviews using current standards of practice in the field and experts in the bioactive–disease outcome relationship. An example of an approach to conducting nutrition systematic reviews is available from the USDA NESR website (23). The review methodology should be appropriate for evaluating nutrition findings because the body of evidence generally differs from placebo-controlled medical or clinical treatment research. Evidence reviewed should focus on human studies representing the population for which the recommendations are intended and can include separate subgroups or a separate review if results could differ by subgroup (e.g., building muscle strength in athletes separately from maintaining lean body mass in sedentary adults).

If the recommendation is intended for the general population, evidence should be based on research conducted in generally healthy people by excluding research designed to treat or reduce symptoms in persons with relevant medical conditions. For example, studies of subjects with diseases known to affect cognitive function (such as multi-infarct dementia and Alzheimer disease) are excluded when evaluating maintenance of normal cognitive function in older adults, and studies of persons with type 2 diabetes are excluded when evaluating maintenance of normal blood glucose after a meal, but not those with elevated glycated hemoglobin at levels deemed prediabetic. Inclusion and exclusion criteria for population, intervention, control, and outcome (PICO) define the scope of the final recommendation.

In the process of conducting the systematic review, bioactive intakes from observational studies reported qualitatively as categories from low to high (e.g., quartiles rather than specific quantities) need to be converted to specific quantities of intake by obtaining that information from the original research investigators. A meta-analysis or pooled study can help determine the amounts (grams per day) of bioactive intake related to the effect. The systematic evidence review of flavan-3-ols and cardiometabolic health by Raman et al. (11) illustrates how to conduct a review for the intended purpose of assessing quantified intake levels of a bioactive with a measurable health outcome.

##### Categorizing the quality of evidence from the systematic evidence review

In addition to quantifying the amount of bioactive intake associated with the health benefit, the evidence should be graded to reflect confidence in the estimated effect of the relationship. Credible up-to-date methods in the field of systematic evidence review practices include but are not limited to GRADE, which rates the quality of evidence based on study design factors, and “risk of bias, imprecisions, inconsistency, indirectness, and magnitude of effect” (21). Other examples include the Agency for Healthcare Research on Quality (commonly referred to a AHRQ) review methodology and the USDA NESR process.

Although the quality of evidence represents a continuum, such processes typically result in assigning evidence into predefined categories. NESR uses 3 quality grades plus “not assignable.” The 2013 GRADE handbook offers a simple transparent way to communicate the quality of evidence according to 4 quality ratings (i.e., very low, low, moderate, and high) reflecting confidence that the “true effect is likely close to the estimate of the effect” (24). When multiple health outcomes or indicator measures are relevant, then each measure should be given a specific quality rating.

### STEP 4: Translate the evidence into a quantified bioactive intake statement

Determine whether the quality of evidence supports making a quantified recommendation. If less than moderate quality, no recommendation is made.
Develop the range of intakes with demonstrated efficacy and safe use.
State the recommendation in this structured form:
  Based on [insert moderate or high] quality evidence, the recommendation is made to consume between [range] grams daily of [dietary bioactive*] to [support structure/function or reduce risk associated with appropriate relationship] among [the general population or specific subpopulation]. *From specific foods if limitations are needed.

#### Determining whether the quality of evidence supports making a quantified recommendation

Using a systematic review with quality of evidence ratings, a panel of experts knowledgeable in both the bioactive and the health outcome quantifies the range of intakes for which there is sufficient quality evidence of a causal relation for normal structure or function, an indicator of health maintenance, or reduced disease risk. Decision-makers should consider that even small effect sizes can have a large impact at a population level. For example, Stamler (25) estimated that reducing population blood pressure by 5 mmHg through an improved dietary sodium/potassium ratio would decrease mortality due to stroke by 14%. Therefore, the standard for minimum effect size for dietary bioactives is suggested to be a measurable clinical change in the direction of a healthier outcome.

When the quality of the evidence is determined to be *less than moderate* (as described in Step 3 of this Framework), the experts should state the decision is to NOT present a recommendation, and provide the rationale regarding data inadequacy with guidance for future research. This step differs from the procedure sometimes used for clinical practice guidelines (21), in which recommendations can be made using relatively lower quality evidence and recommendations that are qualified as “weak” rather than “strong.” The rationale for the simplified construct for bioactives is because dietary recommendations for bioactives are not essential for maintaining health and therefore differ from treatments prescribed to alleviate medical conditions or disease.

Such guidelines for treatment of medical conditions differ in many ways relative to dietary bioactive recommendations for maintaining health, including having a potential for a large magnitude of effect, adverse side effects, higher costs if the condition is not treated, marked changes in quality of life, feasibility, and alternative treatment options. The approach for bioactives summarized in **Table 1** is thus adapted from the GRADE approach to reflect factors relevant to enhanced dietary intake rather than medical treatments. Also, the GRADE categorization into a “no,” “weak,” or “strong” recommendation (26–28) has been modified to eliminate “weak” recommendations; recommendations for bioactives are thus dichotomous: yes/no. In determining whether to quantify a recommended bioactive intake, this bioactive Framework considers the following 2 primary domains:

1. *Confidence in the magnitude of the effect on important outcomes:* The overall quality of the evidence should at least be *moderate* in order to recommend a quantified bioactive intake range.
2. *Balance between desirable and possible undesirable effects:* Beneficial effects should be substantially greater than potential undesirable secondary consequences. Undesirable effects would be relatively unlikely due to this Framework, which sets recommended intakes at or below intakes with a long history of safe use, or (when necessary) values derived from demonstrated experimental safety/toxicology testing. A clear-cut example is that when the range of intakes shown to achieve a benefit exceeds or mostly exceeds the demonstrated history of safe intake, then a quantified intake recommendation for that health outcome is not issued. However, undesirable effects could include factors identified by the expert panel other than “safety,” such as whether intake of energy, nutrients, or recommended food groups is likely to change in an undesirable direction. This could occur, for example, if consuming the specific bioactive in the range recommended would likely contribute excess energy intake in a population with a high prevalence of overweight.

**TABLE 1:** Decide whether the quality of evidence and identified undesirable effects^1^ support making a quantified dietary bioactive intake recommendation based on systematic evidence reviews

Quality of evidence grade	Decision to make a quantified recommendation
Moderate or higher	Yes, contingent on 2 criteria being met:*1*) demonstrated efficacy occurs at levels considered safe, and *2*) bioactive benefits outweigh potential undesirable^1^ effects
Less than moderate	No

1 Undesirable effects identified by the expert panel other than “safety” can include whether intake of energy, nutrients, or recommended food groups are likely to change in an undesirable direction.

Although consumer values, preferences, and costs are among primary considerations for clinical practice guidelines as part of the GRADE approach, they are of relatively less importance for dietary bioactives because individual consumers make those choices on a discretionary basis as part of their everyday food choices. This is unlike medical treatments in which clinical practice guidelines determine the preferred treatment prescribed, taking into consideration important values and preference factors, such as treatment side effects.

#### Setting the range of intake to be recommended

The FNB Guiding Principles report recommended that, rather than specific amounts, ranges be developed for chronic disease risk reduction (4). In 1989, Estimated Safe and Adequate Daily Dietary Intakes (ESADDI) ranges for 8 vitamins and minerals were included in the 10th edition of the FNB's Recommended Dietary Allowances (29). This approach provides the range of intakes over which health benefits are documented; decision-makers who need a single cut-point can use the range to identify an amount that is most relevant to their specific application. The range can be updated as new evidence becomes available.

**FIGURE 2:**
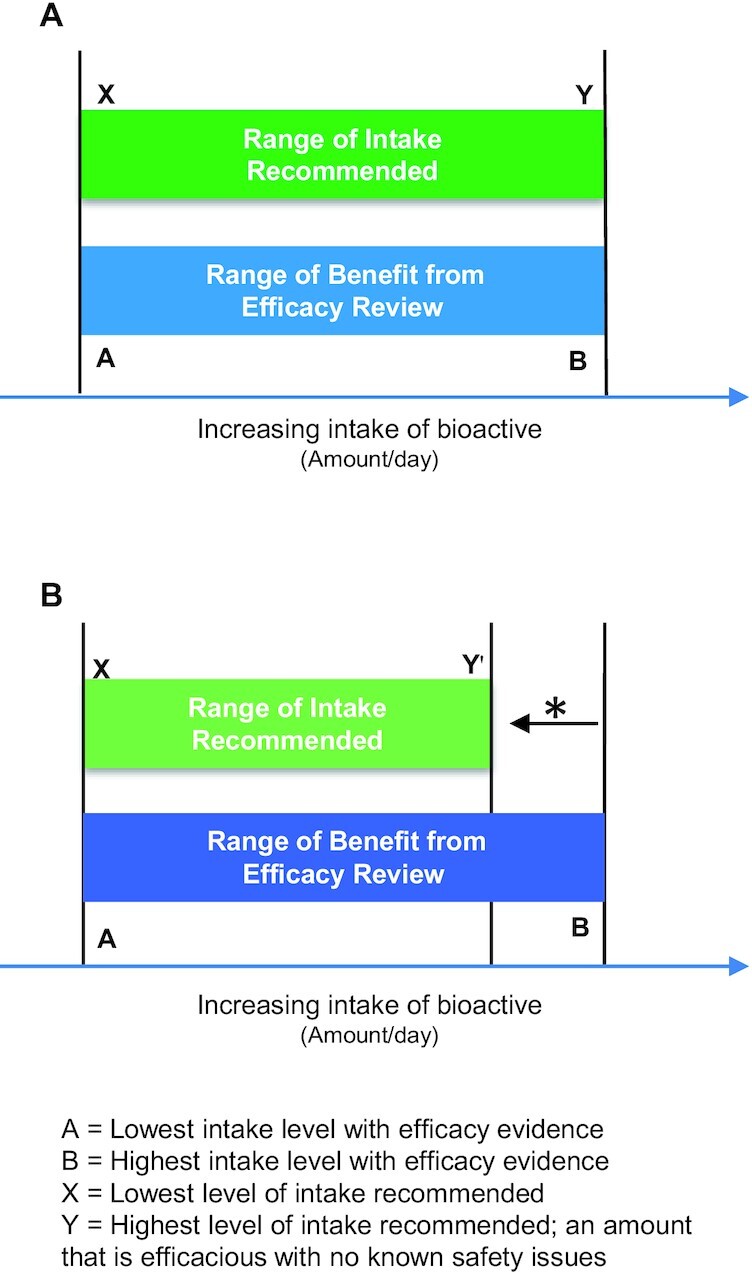
Establishing the recommended intake range for a bioactive. (A) Efficacy intake range *is within known safe range*: the recommended intake range (X to Y) IS SET EQUAL TO the range of demonstrated efficacy (A to B) when this range is *1*) equal to or less than high historical use (e.g., 90th percentile intake) in relevant populations and forms, and *2*) below intake levels with known adverse effects. (B) Efficacy intake range *exceeds known safe intakes*: the upper recommended intake IS ADJUSTED DOWN* to the known safe intake level (Y') based on history of safe use (e.g., 90th percentile intake in a relevant population) and no known adverse effects significant to health.

**Figure 2** shows how to set the recommended range of intake for a bioactive. The lower end of the recommended intake range is the bioactive level of intake with the lowest demonstration of efficacy for maintaining or improving the relevant health outcome (from Step 3). The high end of the recommended range reflects the highest level with demonstrated efficacy, and which does not exceed the highest intake for which no significant adverse health effect is known, based on high historical use levels, published safety literature, or toxicology testing (from Step 2). The range of intake recommended is adjusted down when necessary to an intake level determined to have no adverse effect (from Step 2).

## Operational Considerations in Developing Quantified Recommendations For Bioactives

### Who should develop quantified bioactive intake recommendations?

This Framework is intended to provide guidance to health organizations responsible for developing nutrition recommendations. This includes health professional societies, government agencies, and other nongovernmental authoritative bodies responsible for developing health and nutrition guidance. The Framework is flexible such that it can be adapted to each organization's individual guidance process.

### Role of the health organization(s) in developing recommended intakes

Given the expense and resources needed to properly develop a quantified bioactive intake recommendation, it is important to prioritize the candidate bioactives. Given that diets, risk factors, and health conditions differ greatly from country to country, recommendations developed for one region or country might or might not extrapolate to another specific population. Priorities for developing a quantified bioactive intake recommendation should be informed by experts knowledgeable about the specific country or region to which they will be applied. Two important determinants include: *1*) the purported benefit of the bioactive addresses an unmet need with respect to the major health issues in the population for which recommended intakes would apply (e.g., by country or region), or *2*) the bioactive is heavily marketed or promoted in the media (including social media) to the extent that guidance is needed from the scientific community regarding amounts, if any, that should be recommended.

Integrity and transparency are of primary importance in developing recommended intakes for dietary bioactives. **Box C** highlights key roles of the organization(s) developing recommended bioactive intakes.

Box C: Role of sponsoring organization(s)

1. Ensures that the evidence review(s) is conducted by experts using a transparent and credible recognized systematic approach, and that it
  Operates from a statement of task with a clearly defined scope of work.
  Registers evidence review prior to its conduct.
  Appoints an evidence review committee, which includes both systematic review methodology experts and subject matter experts experienced in the specific bioactive and potential health outcomes.
  Ensures evidence review is published in a peer-reviewed journal, along with detailed grading tables.
2. Oversees recommendation review and development
  Operates expert recommendation panel review from a statement of task with a clearly defined scope of work.
  Appoints the recommendation panel of experts
    With demonstrated expertise (notably as published in peer-reviewed scientific journals) in the bioactive as it relates to the health outcomes under review and in biostatistics and systematic review methodology; and
    Who are free of significant financial, intellectual, and professional conflicts of interest; if such exist, manages and balances bias and conflict of interest according to published policies and procedures (such as is done by NASEM) (30).
3. Identifies and makes publicly transparent a structured guideline development process that is defined before the review begins.
  Oversees an external blinded review by a representative group of scientists and potential users of the report; provides external review comments to the recommendation committee for decisions on final revisions.
  Publishes the recommendation report, with supplementary background information.
4. Ensures financial support is transparent to external entities and in all publications.
5. Establishes plans for periodic re-evaluation.

### Method to ensure periodic re-evaluation of the evidence and update recommendation

The organization(s) responsible for issuing a recommended bioactive intake range and statement should proactively plan for updates with specific timing (e.g., every 5 y) with triggers in place for an earlier review, which could include new safety concerns, new data on bioactive consumption patterns, or new data on efficacy. Proactive steps include:

- Develop an analytical framework for conducting the update.
- Monitor evidence based on a literature scan using keywords from the analytic framework for manuscripts published since the last review. Review abstracts and publications for relevance to prespecified criteria.
- Consult with experts on the bioactive of interest and health outcome(s).
- Establish decision criteria for whether new evidence merits initiating a recommendation update review.

## Conclusion

Quantified ranges of intakes for dietary bioactives are a useful way to accurately relate dietary bioactive consumption with a specific health benefit(s) at levels found to be safe. The step-by-step Framework leading to the structured statement format ensures that all relevant context is communicated along with the recommended intake range, including the form of the bioactive, specific health outcome or indicator measures, and subpopulations affected. Approaches used in developing clinical practice guidelines for medical treatment (including systematic evidence reviews with graded quality of evidence translated into strength of evidence) are modified when quantifying health-promoting dietary bioactive intakes that are consumed as a part of the everyday diet within the context of individual consumer values and preferences.

Translating evidence into recommendations should be conducted using a structured and transparent process managed by credible health organizations experienced in and responsible for developing food and nutrition recommendations. The recommendation panel should rely on members and advisors with strong published scientific expertise in the bioactive and its metabolism, in safety/toxicology, and in the health outcome under review. It should also include input from multiple stakeholders, follow principles of scientific integrity, and be published in a peer-reviewed scientific journal. The resulting dietary bioactive intake recommendations can inform health professionals providing advice to the general public, as well as to scientists conducting research necessary to fill evidence gaps.

## Notes

The International Life Sciences Institute (ILSI), North America Bioactives Committee supported author travel reimbursement. The Institute for the Advancement of Food and Nutrition Sciences (IAFNS) paid publication fees. IAFNS is a public, nonprofit science organization that provides a forum to advance understanding of scientific issues related to the nutritional quality and safety of the food supply. IAFNS receives support primarily from its industry membership.

Author disclosures: AAY is a member of the ILSI-NA and IAFNS Bioactives Committee. JTD is a member of the Scientific Advisory Boards of McCormick Science Institute, Mushroom Council, and Bay State Milling, and a consultant for Motif Foodworks (2019) and Nestlé (2020). JTD holds stock in several food and drug companies; she is editor of *Nutrition Today*, and a member of the ILSI-NA and IAFNS Bioactives Committee. JWE is a Scientific Advisor to the Soy Nutrition Institute, a consultant for Mars, Inc., and a member of the ILSI-NA and IAFNS Bioactives Committee. BJL provides nutrition advice and scientific consultancy to a range of clients in the private for-profit as well as nonprofit food and research sector. She was financially compensated in part for serving on the working group as a consulting senior advisor to the ILSI-NA and IAFNS Bioactives Committee. BOS is a member of the Scientific Advisory Board of McCormick Science Institute and the Board of Trustees of the International Food Information Council. CMW is Scientific Advisor to the California Walnut Commission Health Research Advisory Group (2020–) and California Prune Board Scientific Advisory Group (2019–), and is an ex officio member of the Board of Trustees of ILSI Global (as past Chair). The other author reports no conflicts of interest.

Perspective articles allow authors to take a position on a topic of current major importance or controversy in the field of nutrition. As such, these articles could include statements based on author opinions or point of view. Opinions expressed in Perspective articles are those of the authors and are not attributable to the funder(s) or the sponsor(s) or the publisher, Editor, or Editorial Board of *Advances in Nutrition*. Individuals with different positions on the topic of a Perspective are invited to submit their comments in the form of a Perspective article or in a Letter to the Editor.

Abbreviations used: ADME, absorption, distribution, metabolism, excretion; CDRR, chronic disease risk reduction reference range; FNB, Food and Nutrition Board; GRADE, Grading of Recommendations, Assessment, Development, and Evaluation; IOM, Institute of Medicine; MPOD, macular pigment optical density; NASEM, National Academies of Sciences, Engineering, and Medicine; NESR, Nutrition Evidence Systematic Review; NOFS, nutrient or other food substance; UL, upper level.
